# Enhanced cytotoxicity against cholangiocarcinoma by fifth-generation chimeric antigen receptor T cells targeting integrin αvβ6 and secreting anti-PD-L1 scFv

**DOI:** 10.1186/s12967-025-06453-y

**Published:** 2025-04-16

**Authors:** Nattaporn Phanthaphol, Chalermchai Somboonpatarakun, Kwanpirom Suwanchiwasiri, Pornpimon Yuti, Jatuporn Sujjitjoon, Punn Augsornworawat, George S. Baillie, Mutita Junking, Pa-thai Yenchitsomanus

**Affiliations:** 1 Department of Immunology, Faculty of Medicine Siriraj Hospital, Mahidol University, Bangkok, Thailand; 2 Siriraj Center of Research Excellence for Cancer Immunotherapy (SiCORE-CIT), Research Department, Faculty of Medicine Siriraj Hospital, Mahidol University, Bangkok, Thailand; 3 Division of Molecular Medicine, Research Department, Faculty of Medicine Siriraj Hospital, Mahidol University, Bangkok, Thailand; 4 College of Medical, Veterinary and Life Sciences, University of Glasgow, Glasgow, Scotland, UK

**Keywords:** Cholangiocarcinoma, Solid tumor immunotherapy, Chimeric antigen receptor T cells, Integrin αvβ6, PD-L1 blockade, 3D spheroid model

## Abstract

Cholangiocarcinoma (CCA) is a fatal bile duct cancer with high resistance and recurrence rates, with only one fifth of patients eligible for surgical treatment. The disease resists standard chemotherapy and often relapses. Chimeric antigen receptor (CAR) T cell therapy has shown promise for hematological malignancies but faces challenges in solid tumors due to resistance mechanisms like PD-L1 expression, which tumors use to evade the immune system. To address this challenge, we developed fifth-generation CAR T cells targeting integrin αvβ6 that also secrete anti-PD-L1 single-chain variable fragment (scFv) to target both tumor cells and the PD-1/PD-L1 pathway. We examined integrin αvβ6 and PD-L1 expression in CCA cell lines and engineered T cells to express either fourth-generation CAR T cells targeting integrin αvβ6 (A20 CAR4 T cells) or fifth-generation CAR T cells with anti-PD-L1 scFv secretion (A20 CAR5 T cells). In vitro, A20 CAR5 T cells exhibited less exhaustion and superior long-term functionality compared to A20 CAR4 T cells. In 3D spheroid models of CCA, A20 CAR5 T cells demonstrated enhanced antitumor activity and better infiltration into the spheroid core. These findings suggest that A20 CAR5 T cells have significant potential and warrant further in vivo studies and clinical trials.

**Supplementary Information:**

The online version contains supplementary material available at 10.1186/s12967-025-06453-y.

## Introduction

Cholangiocarcinoma (CCA) is an aggressive bile-duct cancer with a poor prognosis and increasing global incidence, particularly in Northeast Thailand, which has the highest prevalence worldwide [1]. This trend is largely attributed to chronic infection with the liver fluke, *Opisthorchis viverrini* (Ov), and other risk factors, including chronic biliary inflammation such as primary sclerosing cholangitis [2]. Surgical resection remains the only potentially curative option; however, it is feasible in only about 20% of patients, leaving the majority with unresectable tumors. The standard treatment of gemcitabine and cisplatin offers a median overall survival of approximately 12 months [3, 4]. Recently, the addition of durvalumab, an anti-PD-L1 immune checkpoint inhibitor, to this regimen has demonstrated improved survival outcomes [5], underscoring the potential of immunotherapy in CCA. Despite these advances, effective treatment options remain limited, necessitating the exploration of novel therapeutic strategies to improve patient outcomes. Chimeric antigen receptor (CAR) T cell therapy has been approved for the treatment of B cell malignancies and relapsed or refractory multiple myeloma [6, 7]. However, its success in solid tumors, including CCA, has been limited due to challenges such as the immunosuppressive tumor microenvironment and tumor heterogeneity [8, 9]. A previous study targeting two antigens, EGFR and CD133, in resistant CCA showed partial responses, highlighting the potential of multi-targeted CAR T therapy to overcome tumor heterogeneity and improve treatment efficacy [10]. This suggests that developing CAR T cells with enhanced targeting capabilities and resistance to the suppressive tumor microenvironment is crucial for advancing immunotherapeutic strategies in CCA.

Among potential targets, integrin αvβ6 has emerged as a promising candidate due to its selective expression in epithelial cancers, including pancreatic [11], colorectal cancer [12, 13], and CCA [14, 15]. It is highly expressed in approximately 87% of CCA cases and plays a critical role in tumor progression and metastasis [15, 16]. Preclinical studies using blocking antibodies against αvβ6 have demonstrated significant tumor growth inhibition, supporting its therapeutic relevance [13]. However, our previous studies using αvβ6-targeted CAR T cells (A20 CAR4 T cells) revealed that αvβ6 expression level did not directly correlate with effective tumor killing [17], suggesting that additional immunosuppressive mechanisms within the tumor microenvironment may hinder CAR T cell efficacy.

A major mechanism of immune evasion in solid tumors is the upregulation of the PD-1/PD-L1 axis, which induces T cell exhaustion and suppresses anti-tumor immunity [18]. PD-L1 is frequently overexpressed in various solid tumors, including CCA, where it contributes to immune evasion by inhibiting T cell activity [19]. While immune checkpoint inhibitors (ICIs) targeting the PD-1/PD-L1 pathway have shown promise in enhancing tumor responses, their systemic administration is often associated with adverse effects [20, 21]. Combining ICIs with CAR T cells has emerged as a potential strategy to enhance efficacy, but challenges remain in optimizing localized delivery to avoid systemic toxicity.

To address these challenges, we engineered a next-generation CAR T cell therapy (A20 CAR5 T cells) that not only targets integrin αvβ6 but also secretes an anti-PD-L1 single-chain variable fragment (scFv) within the tumor microenvironment. This dual-targeting approach aims to enhance the cytotoxic potential of CAR T cells by simultaneously targeting αvβ6 and neutralizing PD-L1-mediated immunosuppression. We hypothesized that A20 CAR5 T cells would exhibit superior antitumor activity compared to A20 CAR4 T cells by improving tumor infiltration, persistence, and overall cytotoxic efficacy. In this study, we conducted comprehensive in vitro assessments to evaluate the cytotoxic potential, cytokine secretion, and proliferation of A20 CAR5 T cells compared to A20 CAR4 T cells. These analyses were performed using both 2D monolayer cultures and 3D tumor spheroid models to better mimic the in vivo tumor microenvironment and assess the infiltration capabilities of CAR T cells. Our findings provide valuable insights into the potential of next-generation CAR T cell strategies for overcoming immune resistance and improving therapeutic outcomes in CCA.

## Materials and methods

### Cell culture

The patient-derived cholangiocarcinoma cell lines KKU-055 (JCRB1551), KKU-100 (JCRB1568) [35], and KKU-213A (JCRB1557) were sourced from the Japanese Collection of Research Bioresources (JCRB) Cell Bank in Osaka, Japan. These cell lines were maintained in Dulbecco’s Modified Eagle’s Medium (DMEM)/F12 (Gibco; Thermo Fisher Scientific, Waltham, MA, USA) with 10% heat-inactivated fetal bovine serum (FBS, Gibco; Invitrogen) and 100 μg/ml penicillin/streptomycin (Sigma-Aldrich Corporation, St. Louis, MO, USA) at 37 °C in a 5% CO_2_ environment. The Lenti-X^™^ human embryonic kidney (HEK) 293T cells, a subclone of the transformed human embryonic kidney cell line, were obtained from Takara Bio, Inc. (Shiga, Japan). These cells were cultured in DMEM (Gibco; Thermo Fisher Scientific) supplemented with 10% heat-inactivated FBS, 100 U/ml penicillin, and 0.1 mg/ml streptomycin at 37 °C in a 5% CO_2_ atmosphere.

### Flow cytometry

To assess the expression of integrin αvβ6 and PD-L1 proteins on the surface of CCA cells, an anti-integrin αvβ6 antibody (mouse monoclonal, Clone 10D5, Millipore, USA) was used as the primary antibody, followed by a goat anti-mouse Alexa Fluor^®^ 488-labeled secondary antibody (Abcam, Cambridge, MA, USA). For PD-L1 detection, an anti-PD-L1-PE antibody (Clone 29E.2A3; BioLegend, San Diego, CA, USA) was applied.

To evaluate the transduction efficiencies of A20 CAR4 T and A20 CAR5 T cells, CAR surface expression was detected using an anti-c-Myc-FITC antibody (Clone ab1394, Abcam, Cambridge, UK). T cell phenotypic analysis was performed using the following antibodies, all from BioLegend (San Diego, CA, USA): anti-CD3-FITC (Clone UCHT-1), anti-CD4-APC (Clone MEM-241), anti-CD8-APC (Clone UCHT-4), anti-CD16-APC (Clone 3G8), and anti-CD56-PE (Clone AB_2563925).

To determine the binding of secreted anti-PD-L1 scFv, KKU-213A cells were incubated with culture media from NT, A20 CAR4 T, and A20 CAR5 T cells. Binding was detected using anti-HA-Alexa Fluor 647 antibody (Clone 16B2, BioLegend). Flow cytometry was performed using a BD Accuri^™^ C6 Plus Flow Cytometer (BD Biosciences, Franklin Lakes, NJ, USA), and data were analyzed by FlowJo 10.0 software (FlowJo LLC, OR, USA).

### Cell immunofluorescent assay

KKU-055, KKU-100, and KKU-213A cells were seeded on coverslips and cultured overnight. The following day, the cells were rinsed with phosphate-buffered saline (PBS) containing 2% fetal bovine serum (FBS) and fixed with 4% paraformaldehyde on ice. After washing with PBS containing 2% FBS, the cells were blocked with 5% bovine serum albumin (BSA) in PBS for 30 min on ice. The cells were then incubated overnight at 4 °C with mouse anti-integrin β6 antibody (1:100 dilution, Clone 442.5C4; Merck Millipore, Massachusetts, USA) and rabbit anti-PD-L1 antibody (1:100 dilution, Clone 28–8, Abcam, Cambridge, UK). After washes, the cells were incubated with either Alexa Fluor 488-conjugated goat anti-mouse IgG (1:500 dilution, Invitrogen) or Alexa Fluor 555-conjugated swine anti-rabbit IgG (1:500 dilution, Invitrogen) in combination with Hoechst 33342 (1:5,000 dilution, Molecular Probes, Eugene, OR, USA) for nuclear counterstaining. Following a final wash, the coverslips were mounted onto glass slides using a mounting reagent. Imaging was performed using a Nikon Eclipse Ti fluorescence microscope (Nikon, Tokyo, Japan).

### Immunoblot analysis

Immunoblotting was performed to detect PD-L1 expression in CCA cell lines. The cells were lysed in radioimmunoprecipitation assay (RIPA) buffer, and the extracted proteins were separated by 12% sodium dodecyl sulfate–polyacrylamide gel electrophoresis (SDS-PAGE) before being transferred onto a nitrocellulose membrane. The membrane was blocked with 5% skim milk in Tris-buffered saline containing 0.1% Tween-20 (TBST) and then probed with anti-PD-L1 (Clone 28–8, Abcam, Cambridge, UK) and anti-human GADPH (Clone sc-32233, Santa Cruz, Dallas, TX, USA) antibodies.

To assess anti-PD-L1 scFv secretion, the 30 μl of culture supernatants from non-transduced T (NT), A20 CAR4 T cells, and A20 CAR5 T cells were analyzed by SDS-PAGE and immunoblotting. Ponceau S staining was used to confirm equal protein loading. The membrane was blocked with 5% skim milk in TBST and subsequently probed with anti-HA (Clone 2–2.2.14, Invitrogen). Afterward, the membrane was incubated with a horseradish peroxidase (HRP)-conjugated secondary antibody (Invitrogen), and the immunoreaction was visualized using SuperSignal^™^ chemiluminescent substrate (Thermo Fisher Scientific, Waltham, MA, USA). The resulting signal was captured on X-ray film and quantified using ImageJ software (National Institutes of Health, Bethesda, MD, USA).

### Lentiviral vector design and construction

A self-inactivating lentiviral transfer plasmid was constructed to carry the A20 CAR4, following previously established methods [17]. Briefly, the integrin αvβ6-binding peptide (A20) was cloned in-frame with a c-Myc tag sequence linked to the CAR4 sequence. CAR4 consisted of a CD8 short hinge, a CD28 transmembrane domain, three intracellular costimulatory domains (CD28/4-1BB/CD27), and the CD3ζ activation domain (Fig. 2A). Next, cDNA encoding the anti-PD-L1 single-chain variable fragment (scFv), derived from the anti-PD-L1 monoclonal antibody Atezolizumab, was inserted downstream of the A20 CAR4 sequence using *Not*I and *Nhe*I restriction sites. This integration created the A20 CAR4 construct capable of secreting anti-PD-L1 scFv, designated as A20 CAR5. The two components were connected via a T2A peptide, facilitating the cleavage and secretion of the anti-PD-L1 scFv. The entire cDNA sequence was placed under the control of the human elongation factor-1α (*EF-1α*) promoter. The constructed plasmid was then transformed into Stbl3 *Escherichia coli* and extracted using a Midiprep Kit (Qiagen, Hilden, Germany). The accuracy of the inserted gene sequences was confirmed by Sanger sequencing.

### Lentivirus production and CAR T cell generation

To produce lentiviral particles, Lenti-X^™^ HEK293T cells were co-transfected with 30 μg of either the pCDH-EF1α-A20 CAR4 or A20 CAR5 plasmid, along with packaging and envelope plasmids (18 μg of psPAX2 and 6 μg pMD2.G), using a calcium phosphate transfection system. After 12 h, the DMEM was replaced with DMEM supplemented with 5% FBS. Viral particles were harvested at 48- and 72-h post-transfection, filtered through a 0.45 μM filter unit (Millipore, USA) to remove debris, and concentrated by high-speed centrifugation at 20,000 xg for 2 h at 4 °C (Sorvall RC-6 plus centrifuge; Thermo Fisher Scientific, Waltham, MA, USA), and stored at −80 °C for later use. Virus titerd were quantified using a qPCR Lentiviral Titration Kit (Applied Biological Materials [ABM], Richmond, BC, Canada) according to the manufacturer’s protocol.

To generate A20 CAR T cells, peripheral blood mononuclear cells (PBMCs) were isolated from healthy volunteers through density gradient centrifugation using Lymphocyte Separation Medium (Corning, Inc., New York, NY, USA). PBMCs were cultured in AIM-V medium (Gibco, Waltham, MA, USA) supplemented with 5% human AB serum (Sigma-Aldrich) to facilitate the adherence of unwanted monocytes. Non-adherent cells, representing the T cell population, were collected for phenotype analysis. T cells were stimulated with 5 μg/ml phytohemagglutinin-L (PHA-L) (Roche, Basel, Switzerland) for 3 days in AIM-V medium supplemented with 5% human serum, IL-2 (20 ng/ml), IL-7 (10 ng/ml), and IL-15 (10 ng/ml) (Immunotools, Friesoythe, Germany). The PHA-activated T cells were then transduced with lentiviruses in the presence of 10 μg/ml protamine sulfate (Sigma-Aldrich) and spinoculated at 1,200 xg for 90 min at 32 °C. Following transduction, T cells were cultured in medium containing rhIL-2 (20 ng/ml), rhIL-7 (10 ng/ml), and rhIL-15 (10 mg/ml). Flow cytometry was performed at 48- and 72-h post-transduction to assess A20 CAR expression, with further phenotypic analysis conducted on day 6 post-transduction.

### Tumor re-challenge and long-term cytotoxicity assay

A repetitive antigen stimulation assay was conducted in vitro to evaluate the killing efficacy of CAR T cells. KKU-213A cells were co-cultured with non-transduced T cells (NT), A20 CAR4 T cells, or A20 CAR5 T cells at a 5:1 E: T ratio for 72 h (cycle 1). Flow cytometry was used to quantify the remaining viable target cells, and absolute effector cell numbers were determined using counting beads (123count™ eBeads, Thermo Fisher) according to the manufacturer's protocol. Following the first cycle, fresh KKU-213A cells were introduced directly into the existing co-culture without removing the previously seeded cells. This re-exposure process was repeated every three days for a total of three cycles. The sequential tumor rechallenge steps are illustrated in Fig. 3A.

To assess the prolonged cytotoxic potential of A20 CAR4 and A20 CAR5 T cells against integrin αvβ6-positive cells, KKU-213A cells were co-cultured for 6 days (Fig. 3C) at an E: T ratio of 2:1. Cytotoxicity was measured on days 3 and 6 using a crystal violet assay. Following co-culture, T cells were removed, and 100 μl of crystal violet fixing/staining solution was added for 20 min. Excess dye was washed off five times with running water, and the plates were air-dried. Images were captured using an inverted microscope, followed by scanning. The cell bound dye was then solubilized in 100 μl of methanol for 1 h, and absorbance was measured at 595 nm using a Sunrise^™^ absorbance microplate reader with Magellan^™^ data analysis software (version 6.6.0.1; Tecan Group Ltd., Männedorf, Switzerland). Cytotoxicity was calculated using the formula: (1-(absorbance of co-culture with T cells/absorbance of monolayer culture alone)) × 100%.

### 3D tumor spheroid assays

A 3D spheroid model was used to assess the cytotoxicity of A20 CAR T cells. In brief, 4 × 10^3^ cancer cells were mixed with 3% Corning Matrigel matrix (Corning, Inc.) and seeded into an ultra-low attachment 96-well round-bottomed plate (Corning, Inc.). The plate was centrifuged at 1,000 × g for 10 min and incubated for 48 h to allow the formation of a single spheroid. CFSE-labeled T cells (Thermo Fisher Scientific) were added to the well at an E:T ratio of 2:1, along with 2 µg/ml of propidium iodide (PI), for 24, 48 and 72 h. Dead spheroids were analyzed by measuring the increase in PI mean fluorescence intensity (MFI) using a Nikon Eclipse Ti fluorescence microscope (Nikon) and NIS-Elements software. Cytotoxicity was calculated with the formula: [(MFI of treated condition–MFI of spheroid alone)/(MFI of positive control–MFI of spheroid alone)] × 100. The positive control was a spheroid treated with 0.01% Triton-X 100.

A 3D spheroid model was employed to assess T cell infiltration. Specifically, 4 × 10^3^ KKU-213A cells were labeled with CellTracker^™^ Blue 4-chloromethyl-6,8-difluoro-7-hydroxycoumarin (CMF_2_HC), and co-cultured with either NT T cells, A20 CAR4 T cells, or A20 CAR5 T cells, which were labeled with carboxyfluorescein succinimidyl ester (CFSE), at an effector-to-target (E:T) ratio of 2:1. Z-stack images of the co-cultured spheroids were acquired using a confocal microscope to visualize the spatial distribution of T cells within the spheroid. These Z-stack images were then processed using ImageJ to analyze the fluorescence intensity of the CMF_2_HC (blue) signal from the KKU-213A cells and the CFSE (green) signal from the T cells. The fluorescence intensity profiles of the blue CMF_2_HC and green CFSE signals were measured across the Z-stack sections. The intensity profiles for each fluorescence signal were averaged and plotted as histograms (Fig. 6B) to visualize the distribution of both tumor and T cells. To quantify T cell infiltration, only the area under the curve (AUC) for the green CFSE signal was calculated. The AUC represents the total extent of T cell infiltration into the spheroid and was used to compare the level of T cell penetration between the different T cell treatments.

### T cell proliferation assay

To assess the proliferation of CAR T cells in response to CCA cells expressing integrin αvβ6, a carboxyfluorescein succinimidyl ester (CFSE) proliferation assay was performed. Non-transduced T (NT), A20 CAR4 T, or A20 CAR5 T cells were first labeled with CFSE (eBioscience, San Diego, CA, USA). The labeled T cells were then co-cultured with KKU-213A cells at a 5:1 E: T ratio for 3 days without the addition of exogenous cytokines. Following the incubation period, the CFSE-labeled T cells were harvested, and their proliferation was evaluated by measuring the decrease in CFSE fluorescence intensity via flow cytometry.

### Cytokine production assay

Human IFN-γ ELISpot assays were conducted using a commercial kit (Mabtech, AB, Sweden) following the manufacturer's protocol. In brief, PVDF membrane ELISpot plates (Millipore, UK) were pre-coated overnight with an anti-human IFN-γ capture antibody (15 μg/ml) at 4 °C. KKU-213A cells were then co-cultured with CAR T cells at a 5:1 E:T ratio for 24 h. IFN-γ secretion was detected by adding biotinylated anti-IFN-γ detection antibody (1 μg/ml) for 2 h, followed by streptavidin-conjugated alkaline phosphatase (1 μg/ml) for 1 h. Spots were visualized using a BCIP/NBT-plus substrate for 10 min. The plate development was stopped with water, air-dried overnight at room temperature in the dark, and scanned within 24–96 h using a CTL ELISpot reader (Cellular Technology Limited, USA). Spot counts were analyzed using Immunospot 3.1 software.

For cytolytic molecule and cytokine production analysis, NT, A20 CAR4 T, and A20 CAR5 T cells were co-cultured with KKU-213A cells at a 1:2 E:T ratio for 24 h in the cytokine-free medium. After co-culture, supernatants were harvested, centrifuged to remove cellular debris, and stored at -70 °C. Cytokine levels in the supernatants were measured using the LEGENDplex^™^ Human CD8/NK cell panel (#741065, BioLegend) on a BD Accuri^™^ C6 Plus Flow Cytometer, as per the manufacturer's instructions. This panel enables the simultaneous quantification of 13 human cytokines and proteins, including soluble FAS (sFAS), soluble FASL (sFASL), granzyme A, granzyme B, perforin, granulysin, IL-2, IL-4, IL-6, IL-10, IL-17A, IFN-γ, and TNF-α. The data were analyzed using a CytoFLEX flow cytometer (Becton Dickinson (BD) Biosciences, New Jersey, USA).

### Statistical analysis

The experimental results were obtained from at least three independent experiments and are presented as the mean ± standard of deviation (SD) or standard error of the mean (SEM). Comparisons between two groups were performed using a two-tailed *t*-test, while comparisons among three or more groups were analyzed using one-way ANOVA with Bonferroni’s post hoc test. Statistical significance for each comparison was assessed using GraphPad Prism 7 software (GraphPad software, Inc., La Jolla, CA). Differences with a *p*-value of less than 0.05 were considered statistically significant.

## Results

### Expression of integrin αvβ6 and PD-L1 in CCA cell lines

**Fig. 1:**
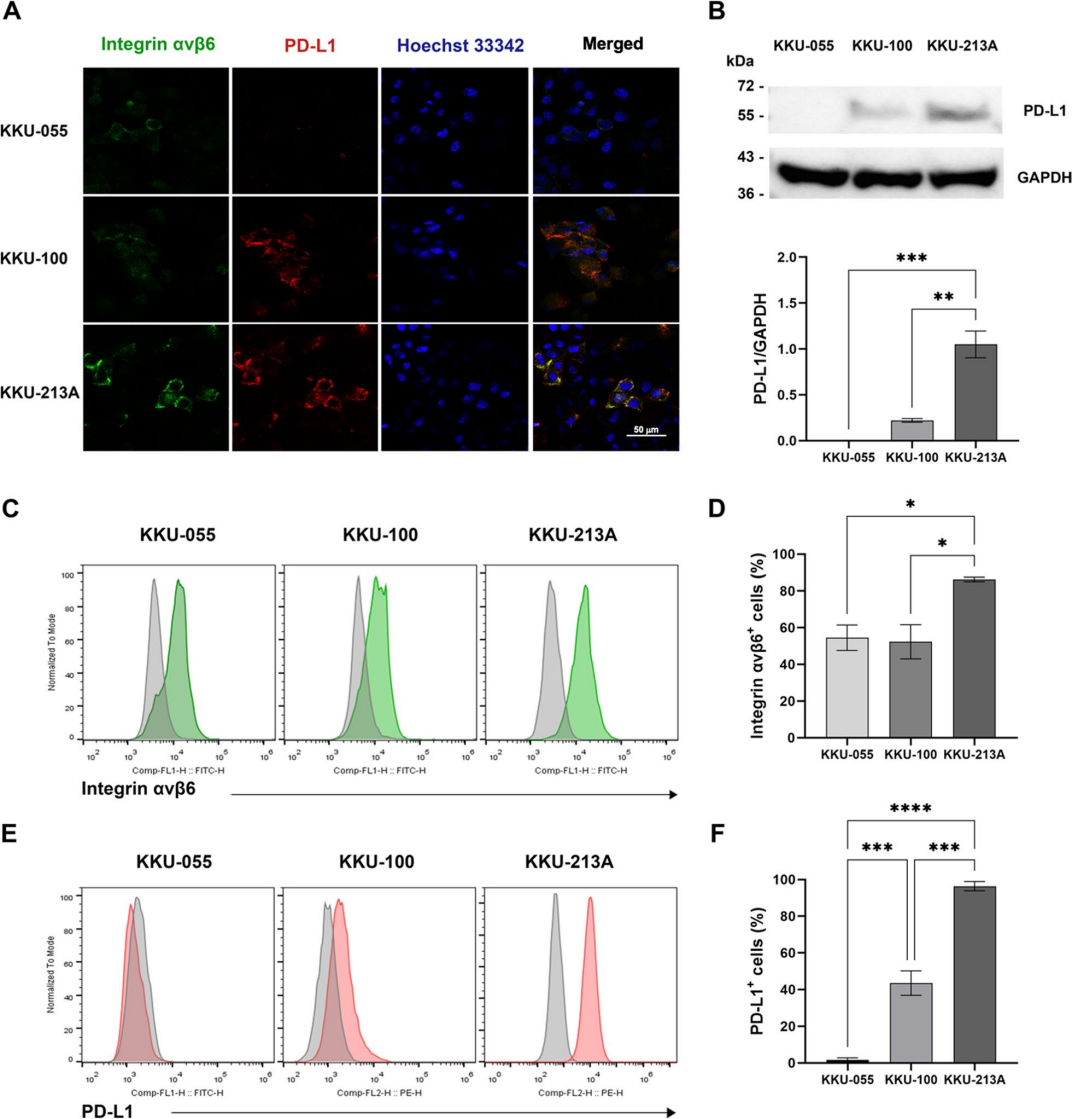
Expression of integrin αvβ6 and PD-L1 in cholangiocarcinoma (CCA) cell lines. **A** Immunofluorescence analysis showing the expression of integrin αvβ6 (green) and PD-L1 (red) in three CCA cell lines: KKU-055, KKU-100, and KKU-213A. The scale bar represents 50 μm. **B** Immunoblot analysis detecting PD-L1 (**~ **55 kDa) and glyceraldehyde 3-phosphate dehydrogenase (GAPDH, 37 kDa) as a loading control (upper panel). Densitometry quantification of PD-L1 relative to GAPDH was performed using ImageJ software (lower panel). **C** Flow cytometry histograms displaying the surface expression of integrin αvβ6 and (**E**) PD-L1, with isotype controls shown in light gray. Quantification of (**D**) integrin αvβ6- and (**F**) PD-L1-positive cells is presented as percentages. Data are shown as mean ± standard error of the mean (SEM) from three independent experiments (N = 3). Statistical analysis was performed using one-way ANOVA with Tukey’s post hoc test (*p < 0.05, ***p < 0.001, ****p < 0.0001)

Building on our previous finding that integrin αvβ6 expression alone was not a reliable predictor of CAR T cell activity targeting integrin αvβ6 [17], we explored the potential role of the immune checkpoint molecule PD-L1 in CCA. PD-L1, known for suppressing T cell function, has been detected in CCA patient tissues [18]. To assess the expression levels of integrin αvβ6 and PD-L1, we performed an immunofluorescence assay on three CCA cell lines: KKU-055, KKU-100, and KKU-213A. Additionally, PD-L1 expression was analyzed using immunoblotting method and flow cytometry. Consistent with our prior observations [17], CCA cells exhibit integrin αvβ6 expression as depicted in Fig. 1A, C–D. The results revealed varying PD-L1 protein levels across the cell lines. Immunofluorescence showed low PD-L1 expression in KKU-055, moderate expression in KKU-100, and high expression in KKU-213A (Fig. 1A). To investigate whether integrin αvβ6 and PD-L1 are co-localized within CCA cells, we performed co-localization immunofluorescence staining, revealing a spatial overlap of integrin αvβ6 and PD-L1 expression in KKU-100 and KKU-213A cells**,** but not in KKU-055 (Fig. 1A, merged images). To validate these results, PD-L1 was further analyzed using immunoblotting (Fig. 1B and Fig. S2A), which confirmed the immunofluorescence findings. Flow cytometry analysis also corroborated these results, revealing low PD-L1 expression in KKU-055 cells (1.7% ± 1.1%), classifying them as αvβ6 ^+^ /PD-L1^−^. Conversely, KKU-100 and KKU-213A cells exhibited significantly higher PD-L1 expression (43.5% ± 6.7% and 96.4% ± 2.6%, respectively; *p* < 0.001) compared to KKU-055 (Fig. 1E, F), classifying them as αvβ6^+^ /PD-L1^+^ . These cell lines were selected based on their integrin αvβ6 and PD-L1 profiles to investigate how these factors interact and impact T cell function in future experiments.

### Generation of A20 CAR4 T and A20 CAR5 T cells and binding capacity of secreted anti-PD-L1 scFv

**Fig. 2:**
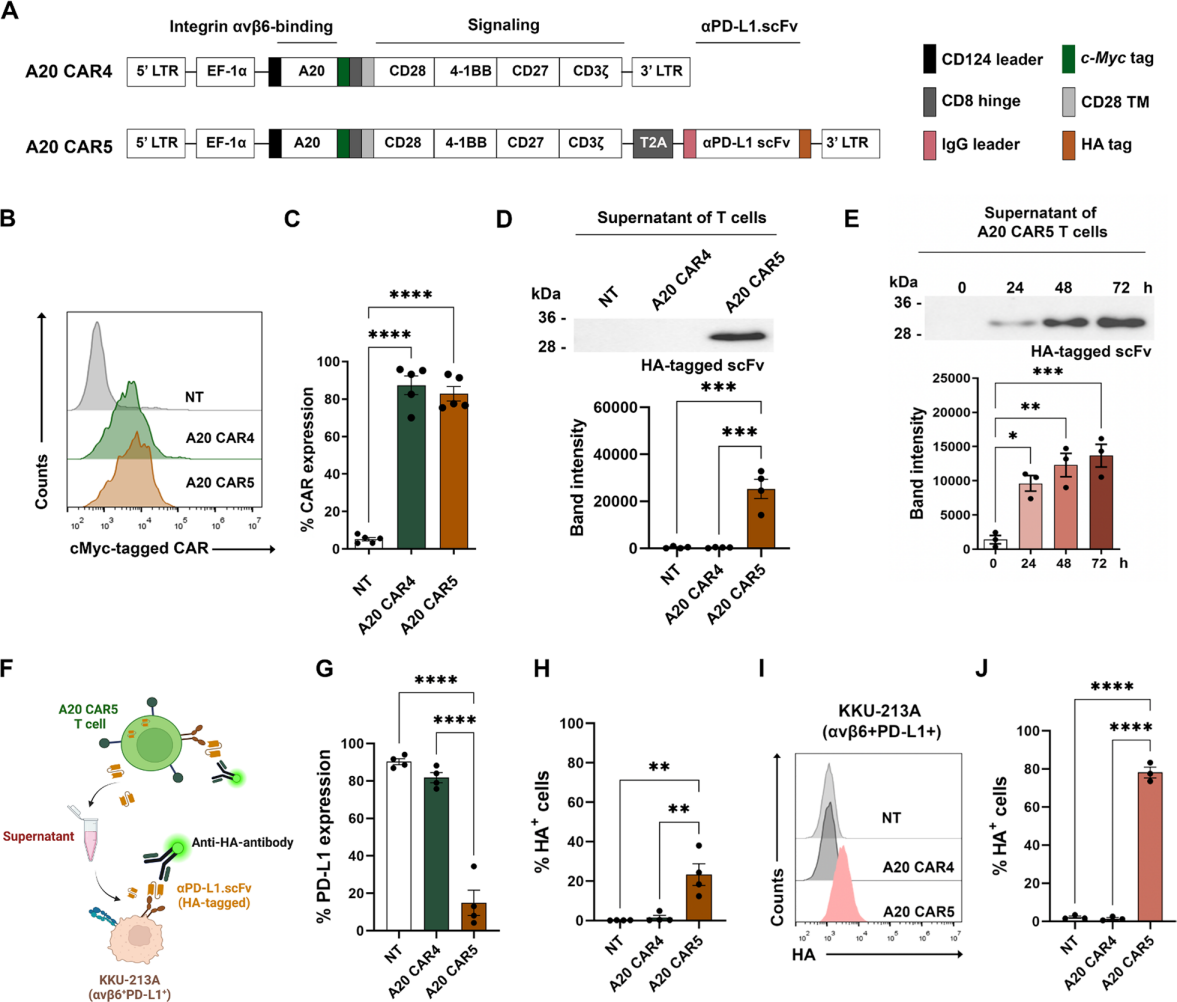
Generation and characterization of A20 CAR4 T and A20 CAR5 T cells. **A** Schematic representations of lentiviral constructs encoding the A20 CAR4 and A20 CAR5 sequences. The original A20 CAR4 construct (upper panel) was modified by adding a cDNA sequence for an anti-PD-L1 scFv at the 3’ end, linked to an HA tag, resulting in the A20 CAR5 construct (lower panel). **B** Histogram plots showing CAR surface expression in primary human T cells detected using an anti-c-Myc antibody. **C** Summary of CAR surface expression data from five independent experiments. **D** Immunoblot analysis of anti-PD-L1 scFv in the supernatants of NT, A20 CAR4 T, and A20 CAR5 T cells cultured for 5 days was detected using an anti-HA tag antibody. **E** Anti-PD-L1 scFv secretion from A20 CAR5 T cells over time: supernatants were collected at 0, 24, 48, and 72 h. **F** Schematic representation of A20 CAR5 T cells secreting anti-PD-L1 scFv, which targets PD-L1 + CCA cells (KKU-213A). **G** PD-L1 expression on effector cells was evaluated after culture. **H** Anti-HA antibody staining was performed to demonstrate the binding of the secreted anti-PD-L1 scFv from A20 CAR5 T cells to PD-L1 on effector cells. **I**, **J** Flow cytometric histograms showing the binding of HA-tagged anti-PD-L1 scFv to KKU-213A cells. The results represent the mean ± standard error of the mean (SEM) from at least three independent experiments using blood samples from different healthy donors. Statistical significance was assessed using one-way ANOVA followed by Tukey’s post hoc test (*p < 0.05, **p < 0.01, ***p < 0.001, ****p < 0.0001)

Our previous study demonstrated the anti-tumor efficacy of A20 CAR4 T cells targeting integrin αvβ6 [17] and the PD-1 and PD-L1 interaction can diminish the efficacy of CAR T cell therapy [22, 23]. Building on this, we engineered CAR T cells to secrete anti-PD-L1 scFv derived from atezolizumab, termed A20 CAR5 T cells. Figure 2A provides a schematic of the lentiviral constructs for A20 CAR4 and A20 CAR5. The A20 CAR4 construct includes cDNA sequences for a signal peptide (CD124 leader), an integrin αvβ6-specific peptide (A20), a c-Myc tag, a CD8 hinge, and CD28 transmembrane and intracellular costimulatory domains (CD28, 4-1BB, CD27), fused to the CD3ζ intracellular activation domain (Fig. 2A, *upper*). The A20 CAR5 construct was derived from A20 CAR4 by adding a cDNA sequence encoding anti-PD-L1 scFv at the 3’-end, linked with an HA tag (Fig. 2A, *lower*). The EF-1α promoter drives both constructs for robust expression in primary human T cells.

This study describes the generation of A20 CAR4 T and A20 CAR5 T cells via lentiviral transduction of primary human T cells with the respective CAR sequences. To facilitate CAR detection, we incorporated a c-Myc tag peptide between A20 and the CD8a hinge domain. This  c-Myc tag enables the detection of c-Myc tagged CAR protein expression on the cell surface, serving as a reliable marker for both CAR expression and transduction efficiency. Flow cytometry analysis confirmed that transduction efficiency, measured by CAR expression, was significantly higher in A20 CAR4 T cells (81.5% ± 7.1%, p < 0.0001) and A20 CAR5 T cells (78.5% ± 5.4%, p < 0.0001) compared to non-transduced (NT) T cells (4.7% ± 0.9%) (Fig. 2B–C). Immunoblot analysis of the supernatants from genetically modified T cells confirmed the presence of secreted anti-PD-L1 scFv, which has an expected molecular weight of about 34 kDa (Fig. 2D and Fig. S2B-C). No HA-tagged protein was detected in the NT and A20 CAR4 T cells, but the supernatant from A20 CAR5 T cells displayed increasing levels of soluble anti-PD-L1 scFv at 24, 48, and 72 h, with adjustments for T cell expansion (Fig. 2E and Fig. S2D–E).

We used flow cytometry to evaluate the binding of secreted anti-PD-L1 scFv to PD-L1 (Fig. 2F) in two scenarios: (1) on the effector T cells themselves and (2) on target KKU-213A cells. Initially, we looked at PD-L1 expression on the effector T cells (Fig. 2G–H). Notably, A20 CAR5 T cells displayed significantly lower PD-L1 detection (14.9 ± 6.7%, *p* < 0.001) when compared to NT (90.3 ± 1.6%) and A20 CAR4 (81.8 ± 2.7%) T cells. Despite this reduced PD-L1 expression, the HA-tagged anti-PD-L1 scFv was found on the surface of A20 CAR5 T cells (23.3 ± 5.5%, *p* < 0.01), indicating successful secretion and binding of the scFv. This binding effectively blocked the detection of monoclonal antibody from binding. Subsequently, we evaluated the binding of the secreted HA-tagged anti-PD-L1 scFv from the culture supernatants of NT, A20 CAR4, and A20 CAR5 T cells to PD-L1 on KKU-213A cells (Fig. 2I, J). Supernatants from A20 CAR5 T cells showed significantly higher binding to PD-L1 on KKU-213A cells (74.8 ± 11.4%, *p* < 0.0001) compared to supernatants from NT (2.7 ± 0.7%) and A20 CAR4 T cells (2.5 ± 0.8%).

### Antitumor effects of A20 CAR5 T cells after prolonging antigen stimulation and tumor re-challenge

**Fig. 3:**
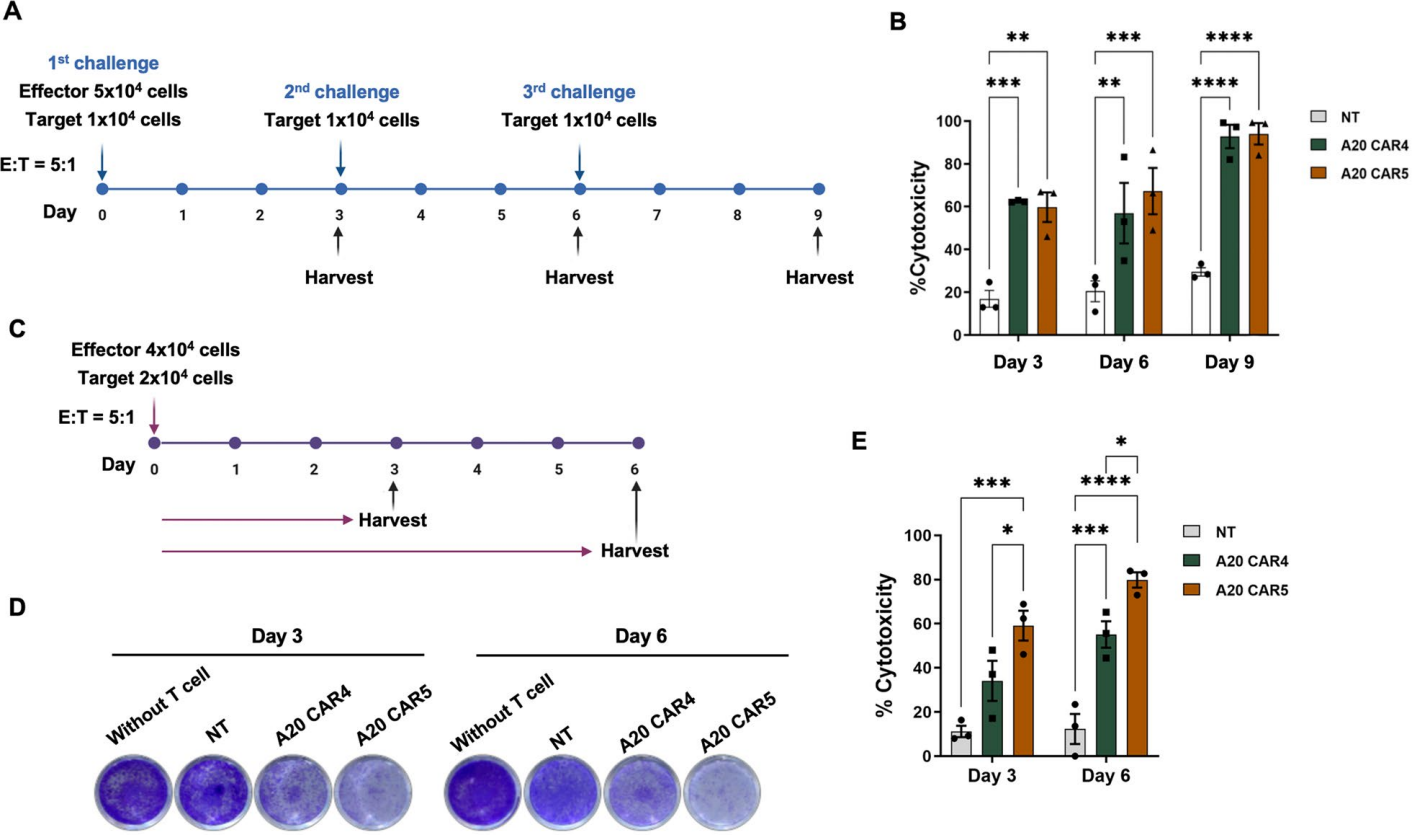
Assessment of the re-challenge killing potential and long-term antitumor efficacy of CAR T cells against KKU-213A cells. **A** Schematic diagram of the re-challenge killing experiment. A20 CAR T cells were co-cultured with KKU-213A target cells at a 5:1 effector-to-target (E:T) ratio. Antigenic stimulation was performed every three days for a total of three cycles over a 9-day period. **B** Cytotoxicity percentages of A20 CAR4 T and A20 CAR5 T cells against target cells were assessed through a flow cytometry-based assay. **C** Timeline and schematic of the long-term antigen challenge, showing CAR T cell proliferation following co-culture with KKU-213A cells at an E:T ratio of 2:1 for 6 days. **D** Cytotoxicity in the long-term antigen challenge assay, assessed via crystal violet staining. **E** Percentage of cytotoxicity by A20 CAR4 T and A20 CAR5 T cells against target cells after prolonged antigen challenge on days 3 and 6. Data from three individual healthy donors are presented as the mean ± SEM. Statistical significance was determined using one-way ANOVA with Tukey’s post hoc test (*p < 0.05, **p < 0.01, ***p < 0.001, ****p < 0.0001)

The cytotoxic potential of NT, A20 CAR4, and A20 CAR5 T cells under conditions of repeated tumor antigen exposure was assessed using a re-challenge assay. This assay involved repeated in vitro co-culture with KKU-213A cells (αvβ6^+^/PD-L1^+)^ at a 5:1 effector-to-target (E:T) ratio. To mimic recurring tumor exposure, fresh tumor cells were added every three days (days 3 and 6), effectively restimulating the T cells. Viable KKU-213A cells were quantified after each 3-day challenge (days 3, 6, and 9; Fig. 3A). NT T cells exhibited consistent, but low, cytotoxicity across all three cycles (17.0 ± 3.9%, 20.5 ± 4.9%, and 29.6 ± 1.9%, respectively). In contrast, both A20 CAR4 and A20 CAR5 T cells demonstrated significantly increased cytotoxicity with each re-exposure compared to NT T cells, reaching 92.8 ± 5.5% (*p* < 0.0001*)* and 94.0 ± 5.0% (*p* < 0.0001*)* on day 9, respectively (Fig. 3B), highlighting their enhanced response to repeated antigen stimulation. No significant difference in cytotoxicity was observed between A20 CAR4 and A20 CAR5 T cells at any time point in this re-challenge setting.

To investigate the sustained anti-tumor efficacy over a longer duration, a distinct long-term co-culture assay was performed. In this assay, A20 CAR4 or A20 CAR5 T cells were co-cultured with KKU-213A cells (αvβ6^+^/PD-L1^+^) at a lower E:T ratio of 2:1 for a total of 6 days without further addition of target cells. Cytotoxicity was assessed on days 3 and 6 (Fig. 3C, D). This experimental design aimed to evaluate the ability of the CAR T cells to control tumor growth over time in the absence of repeated stimulation. Co-culture with NT T cells resulted in consistently low cytotoxicity on both day 3 (11.2% ± 2.6%) and day 6 (12.3% ± 6.8%) (Fig. 3E). In contrast, A20 CAR4 T cells showed significantly higher cytotoxicity by day 6 (55.1% ± 6.0%, *p* < 0.01) compared to NT T cells. A20 CAR5 T cells demonstrated even greater cytotoxicity, significantly higher than NT T cells on day 3 (59.1% ± 6.8% vs. 12.3% ± 6.8%, *p* < 0.05) and A20 CAR4 T cells on day 6 (79.8% ± 3.5% vs. 55.1% ± 6.0%, *p* < 0.001) (Fig. 3E). These results demonstrate a superior and sustained anti-tumor effect of A20 CAR5 T cells compared to both NT and A20 CAR4 T cells in this long-term co-culture assay.

To investigate whether secreted anti-PD-L1 scFv could enhance cytotoxicity at low effector-to-target (E:T) ratios, we collected supernatant from 72-h A20 CAR5 T cell cultures, which contained secreted anti-PD-L1 scFv (Fig. S2). This conditioned medium was then added to a co-culture of A20 CAR4 T cells and KKU-213A cells, and cytotoxicity was compared to that of A20 CAR4 T cells without added supernatant (Fig. S4). The addition of supernatant significantly increased A20 CAR4 T cell cytotoxicity against KKU-213A cells (52.7% ± 12.6%) compared to cells without supernatant (17.8% ± 5.3%, *p* < 0.05). This enhancement was comparable to the cytotoxicity observed with A20 CAR5 T cells, which secrete anti-PD-L1 scFv (50.5% ± 6.2%, *p* < 0.05). Notably, adding the supernatant to A20 CAR5 T cells further boosted cytotoxicity to 77.3% ± 7.3%, higher than A20 CAR5 T cells without supernatant (50.5% ± 6.2%).

### Characterization of A20 CAR T Cells targeting integrin αvβ6- and PD-L1-expressing CCA cells: proliferation, exhaustion, and cytokine/chemokine secretion

**Fig. 4:**
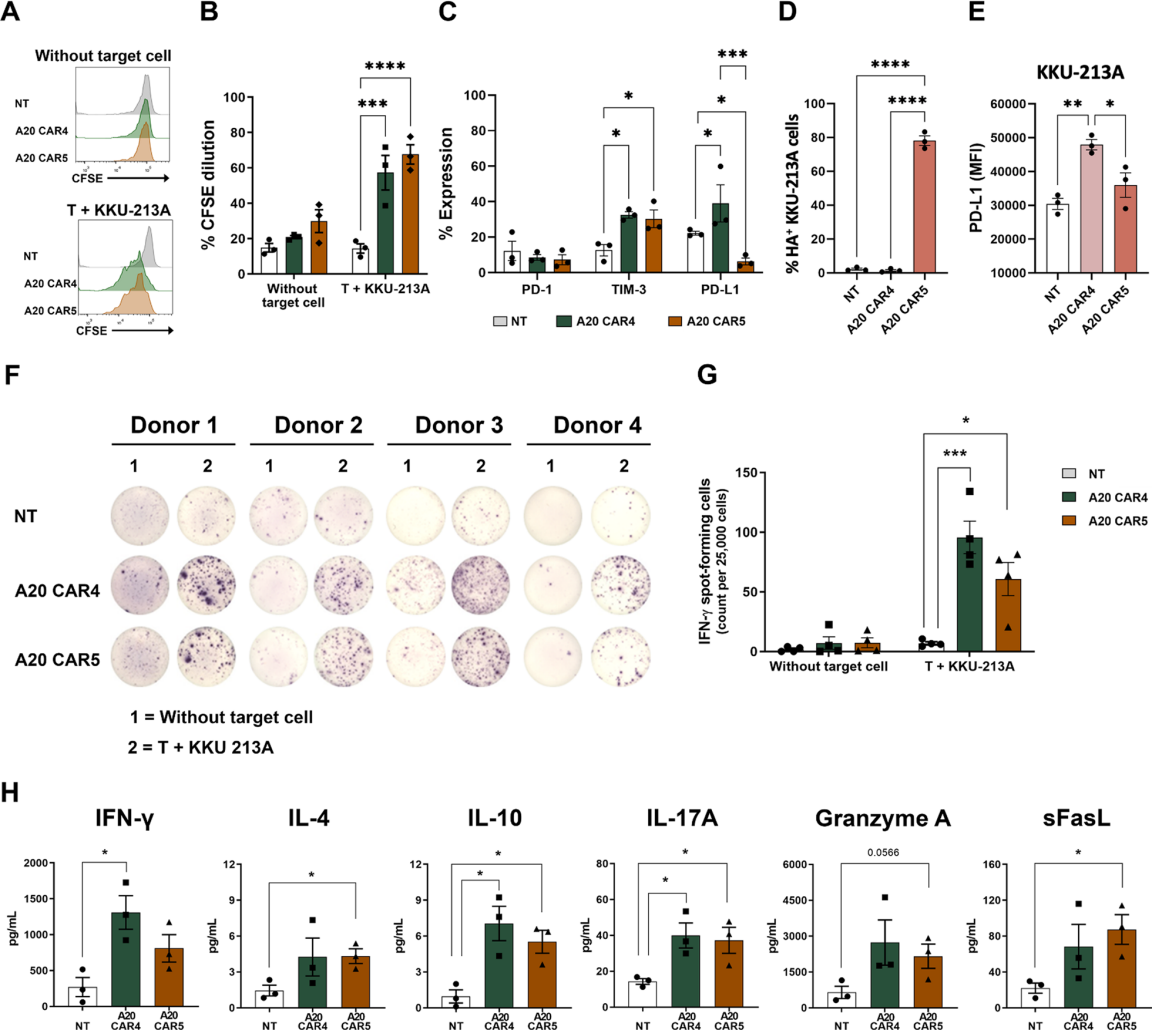
Characterization of A20 CAR T cell response to KKU-213A cells: proliferation, exhaustion, and cytokine/chemokine secretion. **A** Histogram representation and (**B**) percentage of cell proliferation of T cells activated by co-culturing with KKU-213A at an E:T ratio of 2:1 for three days in the absence of exogenous cytokines, examined via CFSE dilution using flow cytometry. **C** Expression levels of exhaustion markers, including PD-1, TIM-3, and PD-L1, were determined in T cells following co-culture with KKU-213A cells for 3 days. **D** Anti-HA antibody staining was performed to assess the binding of secreted anti-PD-L1 scFv from A20 CAR5 T cells to PD-L1 on KKU-213A cells. **E** PD-L1 detection on KKU-213A cells after co-culture was analyzed after co-culture under different conditions: KKU-213A cells alone or exposed to effector T cells (NT, A20 CAR4 T, and A20 CAR5 T cells). The results are presented as the mean ± standard error of the mean (SEM) from at least three independent experiments using blood samples from different healthy donors. **F** Representative picture of ELISpot assay to determine frequencies of IFN-γ spot-forming cells of NT-T, A20 CAR4-T, and A20 CAR5-T cells. **G** IFN-γ spot-forming cells (count per 25,000 cells) from 4 donors. **H** The levels of IFN-γ, IL-4, IL-10, IL-17A, granzyme A and sFasL in the cell culture supernatants of A20 CAR-T cells were analyzed using cytometric bead array (CBA) after 24 h of activation by culturing with KKU-213A cells at an E:T ratio of 5:1. The data were collected from 3 individual healthy donors and presented as mean ± SEM. one-way ANOVA, multiple comparisons were utilized to determine statistical significance (*p < 0.05, and ***p < 0.001)

To evaluate the proliferative capacity of A20 CAR T cells after extended exposure to target cells, a carboxyfluorescein succinimidyl ester (CFSE) proliferation assay was performed on day 3 post-co-culture with KKU-213A cells. The results showed a significant increase in the proliferation rates of A20 CAR4 T (57.3 ± 9.8%, *p* < 0.0001*)* and A20 CAR5 T cells (67.7 ± 5.5%, *p* < 0.0001) compared to NT T cells (14.4 ± 2.6%) (Fig. 4A, B). Additionally, we examined the expression of exhaustion markers (PD-1, TIM-3, and PD-L1) in CAR T cells after long-term stimulation with target cells. Flow cytometry analysis revealed no significant differences in PD-1 expression across all conditions (Fig. 4C). However, TIM-3 expression was markedly higher in A20 CAR4 T cells (32.5 ± 1.8%, *p* < 0.05*)* and A20 CAR5 T cells (30.3 ± 5.0%, *p* < 0.05) compared to NT T cells (12.6 ± 3.2%) (Fig. 4C). Notably, PD-L1 detection exhibited opposite trends: A20 CAR4 T cells showed a significant increase (39.0 ± 10.5%*, p* < 0.05*)*, while A20 CAR5 T cells had a significant decrease (6.3 ± 1.9%, *p* < 0.05) compared to NT T cells (22.2 ± 1.1%) (Fig. 4C). Interestingly, we observed that HA-tagged anti-PD-L1scFv expression on target cells (HA^+^ KKU-213A cells) was significantly higher in co-cultures with A20 CAR5 T cells (78.1 ± 2.8%, *p* < 0.0001) compared to NT T cells (2.17 ± 0.6%) and A20 CAR4 T cells (1.4 ± 0.5%) (Fig. 4D). Additionally, the highest PD-L1 expression was found on the remaining KKU-213A cells co-cultured with A20 CAR4 T cells (47,947 ± 1544) which was significantly greater than the expression observed in target alone (33026 ± 1176, *p* < 0.01), co-cultures with NT T cells (30,413 ± 1,639, *p* < 0.01) and A20 CAR5 T cells (35,976 ± 3633, *p* < 0.05) (Fig. 4E). The upregulation of PD-L1 in response to CAR T cell pressure was particularly notable with A20 CAR4 T cells, which induced a higher level of PD-L1 expression, while A20 CAR5 T cells exhibited lower PD-L1 levels. This difference is likely due to the blocking effect of the anti-PD-L1 scFv, which interferes with the detection of PD-L1 on the target cells in the presence of CAR5.

To profile cytokine production, KKU-213A cells (αvβ6^+^/PD-L1^+^ cells) were co-cultured with NT T, A20 CAR4 T, and A20 CAR5 T cells at an E:T ratio of 5:1. IFN-γ secretion was evaluated using human IFN-γ ELISpot assays. Minimal IFN-γ production was observed in all groups without target cells [NT T: 1.8 ± 1.0, A20 CAR4 T: 7.0 ± 5.3, and A20 CAR5 T cells: 7.3 ± 4.1 IFN-γ spot-forming cells (SFCs) per 25,000 cells]. However, when co-cultured with KKU-213A cells, both A20 CAR4 T and A20 CAR5 T cells showed a significant increase in IFN-γ production (95.7 ± 13.5, *p* < 0.001 and 60.8 ± 13.9, *p* < 0.05 IFN-γ SCFs per 25,000 cells, respectively), compared to NT T cells (6.8 ± 1.3 IFN-γ SFCs per 25,000 cells,) (Fig. 4F–G).

Additionally, cytokine levels were measured after a 24-h co-culture with KKU-213A cells using the LEGENDplex™ Human CD8/NK Cell Panel Cytokine Bead Array (CBA). A20 CAR4 T cells secreted significantly higher amounts of IFN-γ (1309.0 ± 403.8 pg/ml, *p* < 0.05), IL-10 (7.1 ± 1.4 pg/ml, *p* < 0.05), and IL-17A (40.0 ± 12.1 pg/ml, *p* < 0.05) compared to NT T cells (269.3 ± 230.6 pg/ml, 1.0 ± 0.6 pg/ml, and 14.3 ± 1.6 pg/ml, respectively) (Fig. 4F). Although A20 CAR5 T cells also exhibited significantly elevated levels of IL-10 (5.5 ± 1.0 pg/ml, *p* < 0.05) and IL-17A (37.2 ± 7.2 pg/ml, *p* < 0.05) compared to NT T cells, these levels were not statistically different from those of A20 CAR4 T cells. Notably, A20 CAR5 T cells demonstrated increased production of granzyme A (2160.0 ± 508.3 pg/ml, *p* < 0.05) and sFasL (87.3 ± 16.6 pg/ml, *p* < 0.05) compared to NT T cells (649.8 ± 254.8 pg/ml and 22.0 ± 5.7 pg/ml, respectively) (Fig. 4F). These findings suggest that both A20 CAR4 T and A20 CAR5 T cells exhibit enhanced activation and lytic potential against integrin αvβ6-positive CCA cells.

### Efficacy of A20 CAR T cells in eliminating and infiltrating integrin αvβ6 and PD-L1-expressing CCA cells in spheroid culture

**Fig. 5:**
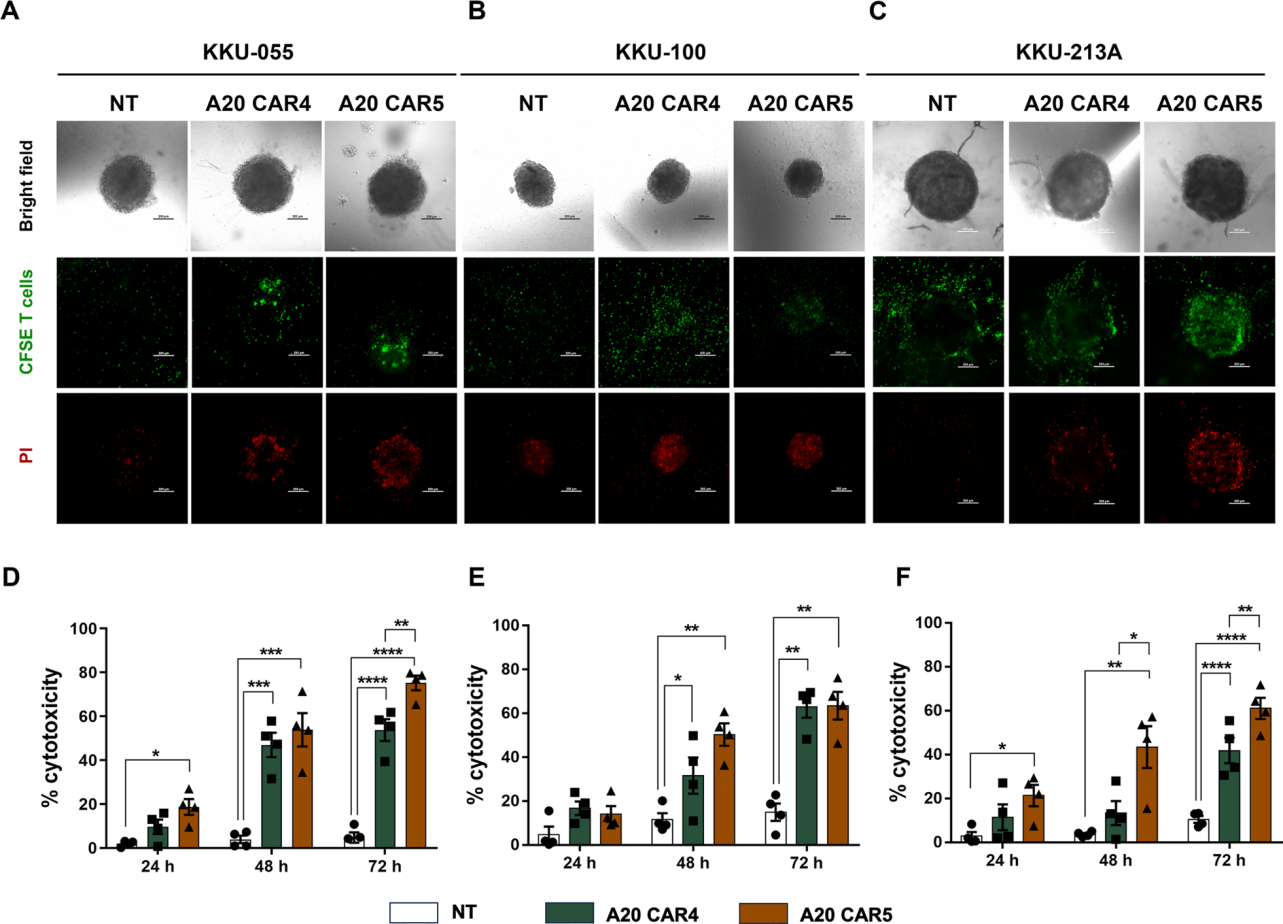
Cytotoxic efficacy of A20 CAR T cells against CCA cell lines in a 3D spheroid model. Confocal microscopy images illustrate the cytotoxic effects of NT T, A20 CAR4 T, and A20 CAR5 T cells on (**A**) KKU-055 (αvβ6^+^/PD-L1^−^), (**B**) KKU-100 (αvβ6^+^/PD-L1^+^), and (**C**) KKU-213A (αvβ6^+^/PD-L1^+^) spheroids. Red fluorescence indicates dead cells, as detected by propidium iodide (PI) uptake, following co-culture with green CFSE-labeled CAR T cells for 72 h at an E:T ratio of 2:1. Scale bar = 200 μm. **D**–**F** Cytotoxic activity was evaluated over 24, 48, and 72 h by measuring PI incorporation to quantify cell death within spheroids. The efficacy of NT T cells, A20 CAR4 T cells, and A20 CAR5 T cells was compared. Cytotoxicity percentages were calculated relative to a positive control induced by 0.01% Triton-X 100. Data were collected from four individual healthy donors and are presented as mean ± SEM. Statistical significance was determined using one-way ANOVA followed by Tukey’s post hoc test (*p < 0.05, **p < 0.01, ***p < 0.001, ****p < 0.0001)

The constraints of traditional two-dimensional (2D) cell cultures, where cells grow on flat surfaces, prompted us to use three-dimensional (3D) cholangiocarcinoma (CCA) spheroids, as they more accurately replicate the intricate interactions between cells and their extracellular matrix present in solid tumors. To evaluate the efficacy of A20 CAR T cells, we generated these spheroids by embedding KKU-055, KKU-100, and KKU-213A cells in Matrigel for 48 h. We then co-cultured the spheroids with CFSE-labeled NT T, A20 CAR4 T, and A20 CAR5 T cells, followed by staining with propidium iodide (PI) to detect dead cells. Confocal microscopy at 24, 48, and 72 h revealed that spheroids co-cultured with NT T cells showed minimal change, with green fluorescence indicating the persistence of target cells. In contrast, spheroids co-cultured with A20 CAR T cells displayed red PI-stained regions, signaling cell death (Fig. 5A–C). Cytotoxicity was quantified by measuring the mean fluorescence intensity of PI staining in spheroids under different conditions. A20 CAR T cells exhibited time-dependent cytotoxicity across all three CCA spheroids (KKU-055, KKU-100, KKU-213A), surpassing that of NT T cells (Fig. 5A–C). In particular, A20 CAR5 T cells demonstrated significantly greater cytotoxicity against KKU-055 spheroids compared to both NT T cells and A20 CAR4 T cells. At 24, 48, and 72 h, A20 CAR5 T cells produced cytotoxicity rates of 18.7 ± 3.6% (*p* < 0.05), 53.9 ± 7.6% (*p* < 0.001), and 75.2 ± 3.3% (*p* < 0.01), respectively, compared to NT T cells (2.0 ± 0.6%, 3.9 ± 1.7%, and 4.8 ± 2.3%, respectively), and A20 CAR4 T cells at 72 h (75.2 ± 3.3% vs. 53.8 ± 5.0%, *p* < 0.01) (Fig. 5D). Similar trends were observed for KKU-100 spheroids, with A20 CAR5 T cells and A20 CAR4 T cells showing higher killing activity than NT T cells at 48 and 72 h (Fig. 5E). In KKU-213A spheroids, A20 CAR5 T cells demonstrated significantly greater cytotoxicity compared to non-transduced (NT) T cells at 24 h (21.3% ± 4.9% vs. 2.8% ± 1.9%, *p* < 0.05), 48 h (43.4% ± 9.5% vs. 3.7% ± 0.6%, *p* < 0.01), and 72 h (61.1% ± 4.9% vs. 10.5% ± 1.6%, *p* < 0.0001). Furthermore, A20 CAR5 T cells exhibited significantly higher cytotoxicity than A20 CAR4 T cells at 48 h (43.4% ± 9.5% vs. 13.3% ± 5.5%, *p* < 0.05) and 72 h (61.1% ± 4.9% vs. 41.8% ± 5.8%, *p* < 0.01) (Fig. 5F). Additionally, in KKU-055 cells overexpressing PD-L1 following IFN-γ induction (Fig. S3A), only A20 CAR5 T cells exhibited sustained and robust cytotoxicity, achieving a killing efficacy of 52.2 ± 10.7%, *p* < 0.01. In contrast, NT T cells demonstrated minimal cytotoxicity, with a killing rate of only 1.3 ± 0.8%. A20 CAR4 T cells, while still showing some activity, exhibited significantly reduced killing efficacy (20.9 ± 6.2%, *p* < 0.05) compared to A20 CAR5 T cells, highlighting the enhanced functionality of A20 CAR5 T cells in overcoming PD-L1-mediated inhibition (Fig. S3B-C).

**Fig. 6:**
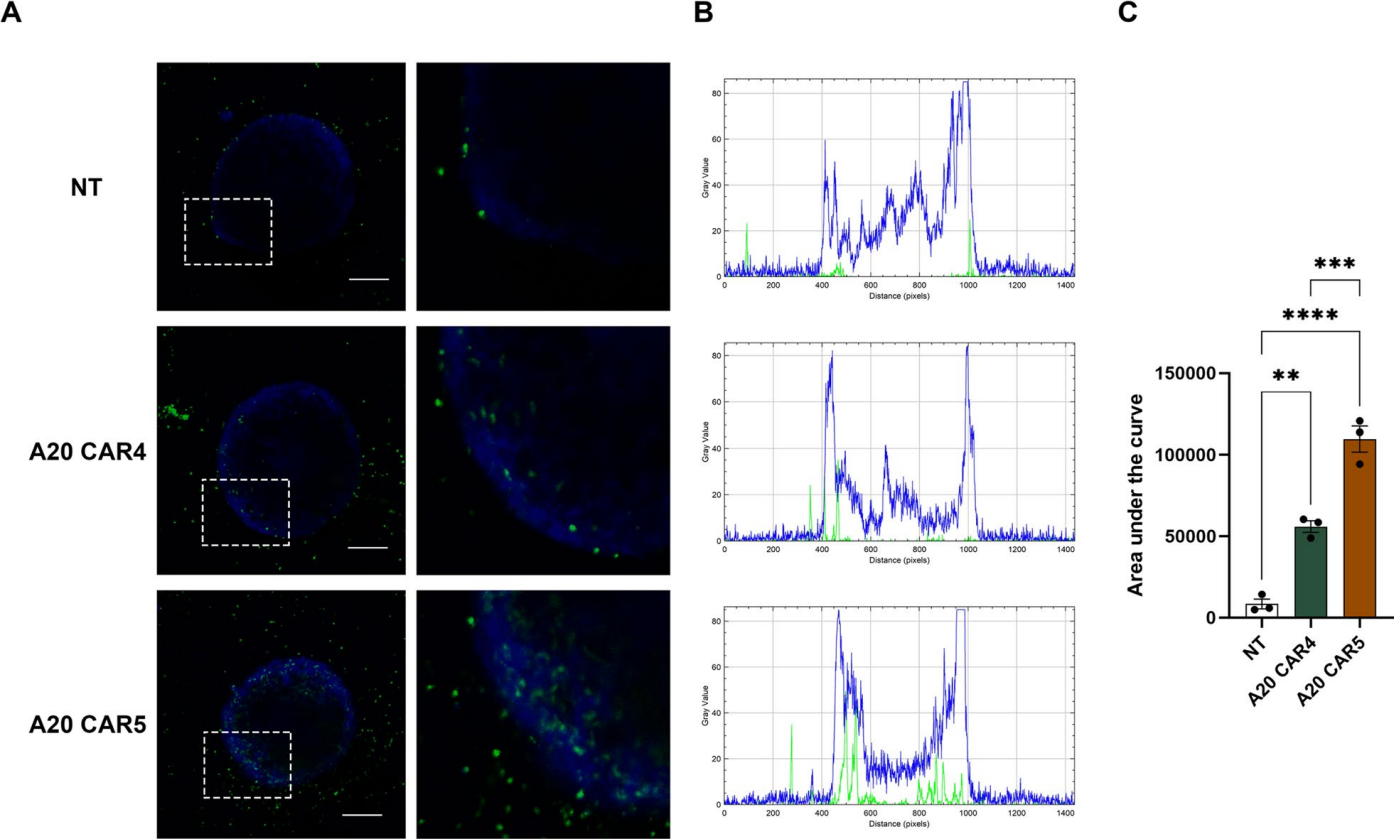
A20 CAR5 T cells infiltrate integrin αvβ6^+^/PD-L1^+^ KKU-213A spheroids. **A** Representative images were captured to visualize the infiltration of A20 CAR T cells into the spheroids. In brief, 4 × 10^3^ cells of KKU-213A labeled with CellTracker^™^ Blue 4-chloromethyl-6,8-difluoro-7-hydroxycoumarin (CMF_2_HC) were co-cultured with either NT T, A20 CAR4 T or A20 CAR5 T cells labeled with CFSE (Thermo Fisher Scientific) at an E:T ratio of 2:1. **B** Histograms represent the averaged intensity profiles of blue CMF_2_HC KKU-213A spheroids and green CFSE T cells signals in z-stack cross-sections of spheroids defied by ImageJ. **C** T cell infiltration was quantified by calculating the area under the curve (AUC) of the green CFSE signals. Scale bar = 200 μm. Statistical significance was determined using one-way ANOVA followed by Tukey’s post hoc test (**p < 0.01, ***p < 0.001, ****p < 0.0001)

To investigate CAR T cell infiltration, tumor spheroids were cocultured with either NT, A20 CAR4 T, or A20 CAR5 T cells. Z-stack images were captured using confocal microscopy to visualize the tumor spheroids labeled with blue CMF_2_HC and the T cells labeled with green CFSE. The images were then analyzed using ImageJ to quantify T cell infiltration into the spheroids (Fig. 6). To evaluate T cell infiltration, we measured the fluorescence intensity of the green CFSE signal within the spheroid region. Both A20 CAR4 and A20 CAR5 T cells demonstrated significantly higher infiltration into the spheroid compared to NT T cells, as indicated by the increased green fluorescence intensity observed within the blue spheroid (Fig. 6A). Notably, A20 CAR5 T cells exhibited deeper penetration into the inner core of the spheroid than A20 CAR4 T cells, with this difference reaching statistical significance (*p* < 0.001) (Fig. 6B, C). Quantification of fluorescence intensity along the z-axis further confirmed this trend, showing stronger green fluorescence signals in the deeper regions of the spheroid for A20 CAR5 T cells, suggesting enhanced infiltration capability.

## Discussion

Preclinical studies with A20 CAR T cells, which target integrin αvβ6 in CCA, have also demonstrated efficacy [17]. Treating solid tumors like CCA with CAR T cells presents unique challenges due to the immunosuppressive tumor microenvironment (TME). Thus, disrupting the PD-1/PD-L1 pathway is essential to improving CAR T cell effectiveness, especially in tumors with high PD-L1 expression. In this study, we aim to address this challenge by engineering fourth-generation CAR T cells targeting integrin αvβ6 (A20 CAR4 T cells) and enhancing their function by incorporating the secretion of anti-PD-L1 scFv (A20 CAR5 T cells). This anti-PD-L1 scFv disrupts the PD-L1/PD-1 interaction, potentially leading to increased tumor cytotoxicity and improved CAR T cell performance in CCA.

The upregulation of integrin αvβ6 in CCA is associated with poor patient prognosis [15] and may contribute to immune evasion, as observed in head and neck cancer [26]. In our study, we analyzed the expression of integrin αvβ6 and PD-L1 in three human CCA cell lines: KKU-055, KKU-100, and KKU-213A. All three cell lines expressed integrin αvβ6. However, KKU-055 cells showed low levels of PD-L1, whereas KKU-100 and KKU-213A cells demonstrated higher PD-L1 expression (Fig. 1). Co-localization immunofluorescence staining revealed a spatial overlap between integrin αvβ6 and PD-L1 in KKU-100 and KKU-213A, suggesting a potential tumor microenvironment-dependent regulation of these proteins. This finding is significant because elevated PD-L1 levels have been associated with poorer patient survival in CCA [18, 28]. PD-L1 is emerging as both a therapeutic target and a biomarker for predicting response to immunotherapy in CCA [25, 30]. Given that both integrin αvβ6 and PD-L1 have been separately linked to poor prognosis in CCA**,** further investigation into their co-expression in clinical samples and association with patient survival is warranted. Understanding whether their combined presence influences tumor progression, immune suppression, or therapeutic response could help in refining patient selection for integrin αvβ6- and PD-L1-targeting therapies**.**

To further investigate the relationship between these molecules, we conducted bioinformatics analysis using publicly available datasets (GSE132305, GSE26566, and GSE32225) (Fig. S1). Surprisingly, our analysis revealed no significant correlation between *ITGB6* and *CD274* (PD-L1) expression at the transcriptomic level in CCA**.** This suggests that while both proteins may be independently upregulated in CCA and contribute to immune evasion, their expression is not necessarily co-regulated at the mRNA level. This discrepancy highlights the complex regulatory mechanisms governing PD-L1 expression**,** which may be influenced by epigenetic modifications, cytokine signaling, or post-transcriptional regulation rather than direct co-expression with integrin αvβ6. These results highlight the importance of developing multi-targeted immunotherapeutic approaches to overcome immune suppression in CCA**.**

To improve the effectiveness of our previous A20 CAR4 T cell therapy, which targeted integrin αvβ6 to eliminate CCA cells [17], we engineered A20 CAR5 T cells to co-express A20 CAR4 and secrete anti-PD-L1 scFv, addressing the PD-1/PD-L1 checkpoint pathway (Fig. 2A). After generating both A20 CAR4 T and A20 CAR5 T cells, we confirmed successful transduction by analyzing CAR protein expression in both populations (Fig. 2B–E and S2). Importantly, the inclusion of anti-PD-L1 scFv did not affect transduction efficiency, demonstrating the feasibility of multi-functional CAR T cell designs. Our data further demonstrated that the secreted anti-PD-L1 scFv effectively binds to PD-L1 on both effectors (Fig. 2F–H) and cancer cells (Fig. 2I, J). Interestingly, preincubation with the secreted anti-PD-L1 scFv specifically bound to PD-L1 at the overlap epitope recognized by the detecting anti-PD-L1 monoclonal antibody. These findings demonstrate that the PD-1/PD-L1 interaction can be partially reversed through blockade mediated by the secreted anti-PD-L1 scFv from A20 CAR5 T cells. This approach aligns with the growing evidence supporting synergistic effects when combining CAR T cell therapy with immune checkpoint blockades (ICBs). Preclinical and clinical studies have demonstrated that ICBs enhance CAR T cell infiltration and persistence, particularly when using anti-PD-1 [30] or anti-PD-L1 antibodies [31] in combination with CAR T cell therapy.

A major challenge in CAR T cell therapy is the potential for functional decline and disease progression due to chronic antigen exposure, which can lead to T cell exhaustion and relapse [32]. Chronic antigen exposure can induce CAR T cell exhaustion, characterized by diminished cytotoxicity, proliferation, and cytokine production, potentially resulting in relapse. While A20 CAR4 and CAR5 T cells demonstrated comparable cytotoxicity against KKU-213A cells in a serial exposure model (Fig. 3A, B), the superior persistence of A20 CAR5 T cells suggests a potential for sustained antitumor activity. Notably, the continued proliferation of CAR T cells led to an increased effector-to-target (E:T) ratio by day 6, which likely contributed to the enhanced cytotoxicity observed at the 9-day time point (Fig. 3B). This observation underscores the ability of CAR T cells to maintain tumor clearance over prolonged antigen exposure. Importantly, the reduced cytotoxicity of A20 CAR4 T cells could be restored by adding supernatants containing secreted anti-PD-L1 scFv from A20 CAR5 T cells (Fig. S4), resulting in sustained long-term killing even at low effector-to-target ratios (Fig. 3C–E). Additionally, both A20 CAR4 and A20 CAR5 T cells demonstrated enhanced function, likely attributed to their sustained proliferation (Fig. 4A, B).

To better understand the underlying mechanisms, we examined exhaustion markers. While PD-1 expression was consistent across all T cell types, we observed distinct patterns of TIM-3 and PD-L1 expression. A20 CAR4 T cells exhibited higher levels of TIM-3 and PD-L1, whereas A20 CAR5 T cells had significantly reduced PD-L1 detection (Fig. 4C). The reduced PD-L1 detection on A20 CAR5 T cells is likely due to steric hindrance from the secreted anti-PD-L1 scFv, which interferes with monoclonal antibody binding rather than an actual reduction in PD-L1 expression. This was supported by the presence of HA-tagged anti-PD-L1 scFv on target cells in co-culture with A20 CAR5 T cells and the significantly lower PD-L1 levels detected in these conditions (Fig. 4D, E). Additional validation through SDS-PAGE/WB may provide further confirmation. However, PD-L1 is also highly expressed in immunosuppressive cells within the tumor microenvironment, such as macrophages and myeloid-derived suppressor cells (MDSCs) [33, 34]. Previous research has shown that CAR T cells alone may struggle to overcome this immunosuppressive environment, highlighting the potential advantages of combining CAR T therapy with PD-L1 blockade to fully unlock their antitumor capabilities [35].

CAR T cells typically secrete proinflammatory cytokines such as IL-2, TNF-α, and IFN-γ, which play crucial roles in regulating cell growth, activation, and differentiation [36]. The addition of 4-1BB has been shown to significantly increase Th1 cytokines like IFN-γ and GM-CSF, while CD28 inclusion leads to higher levels of IL-10 and IL-4 [37]. In our study, both A20 CAR4 T and A20 CAR5 T cells exhibited significantly higher secretion of IFN-γ compared to NT T cells, indicating a strong antigen-specific response (Fig. 4F–G). Additionally, CAR T cells rely on granule-mediated apoptosis to destroy tumor cells. Both A20 CAR4 T and A20 CAR5 T cells secreted elevated levels of granzyme A compared to NT T cells (Fig. 4H). Notably, A20 CAR5 T cells also triggered higher levels of IL-4, IL-10, IL-17A, and soluble Fas ligand (sFasL) after co-culture with KKU-213A cells (Fig. 4F). IL-17A is known to play a key role in controlling tissue infections by promoting the expression of pro-inflammatory cytokines and chemokines, while IL-4, referred to as the "master immune-stimulating cytokine," regulates antibody production, hematopoietic and inflammatory responses, and effector T cell responses [38]. Moreover, CAR T cells can eliminate antigen-negative tumor cells in an antigen-positive environment via the Fas/FasL pathway [39]. The increased sFasL levels observed in A20 CAR5 T cells, compared to NT T cells, may further enhance their cytotoxic activity. Interestingly, the secretion of anti-PD-L1 scFv from A20 CAR5 T cells did not alter the cytokine release profile triggered by αvβ6-positive target cells. This indicates that integrin αvβ6-targeted CAR stimulation promotes a balanced Th1/Th2 cytokine response, specific to the targeted antigen.

Cholangiocarcinoma (CCA) creates a tumor microenvironment (TME) characterized by T cell exclusion and immune evasion, hindering effective immune responses. While T cell infiltration into CCA tissue is associated with improved patient outcomes [40], preclinical evaluation of immunotherapies requires models that recapitulate the complex TME. Therefore, we utilized a Matrigel-based 3D spheroid culture system, which provides a more realistic representation of the in vivo extracellular matrix and TME compared to conventional 2D monolayers [41], to assess the cytotoxicity of A20 CAR T cells. Our results demonstrate that A20 CAR5 T cells significantly outperformed A20 CAR4 T cells in eradicating PD-L1-high 3D cancer spheroids. Specifically, A20 CAR5 T cells exhibited superior cytotoxicity against highly PD-L1-expressing KKU-213A cells, demonstrating enhanced infiltration into the spheroid core. These findings highlight the superior efficacy of A20 CAR5 T cells in targeting and penetrating integrin αvβ6^+^/PD-L1^+^ tumor spheroids, suggesting their potential as a promising therapeutic approach for solid tumors (Figs. 5–6 and Fig. S3).

**Fig. 7:**
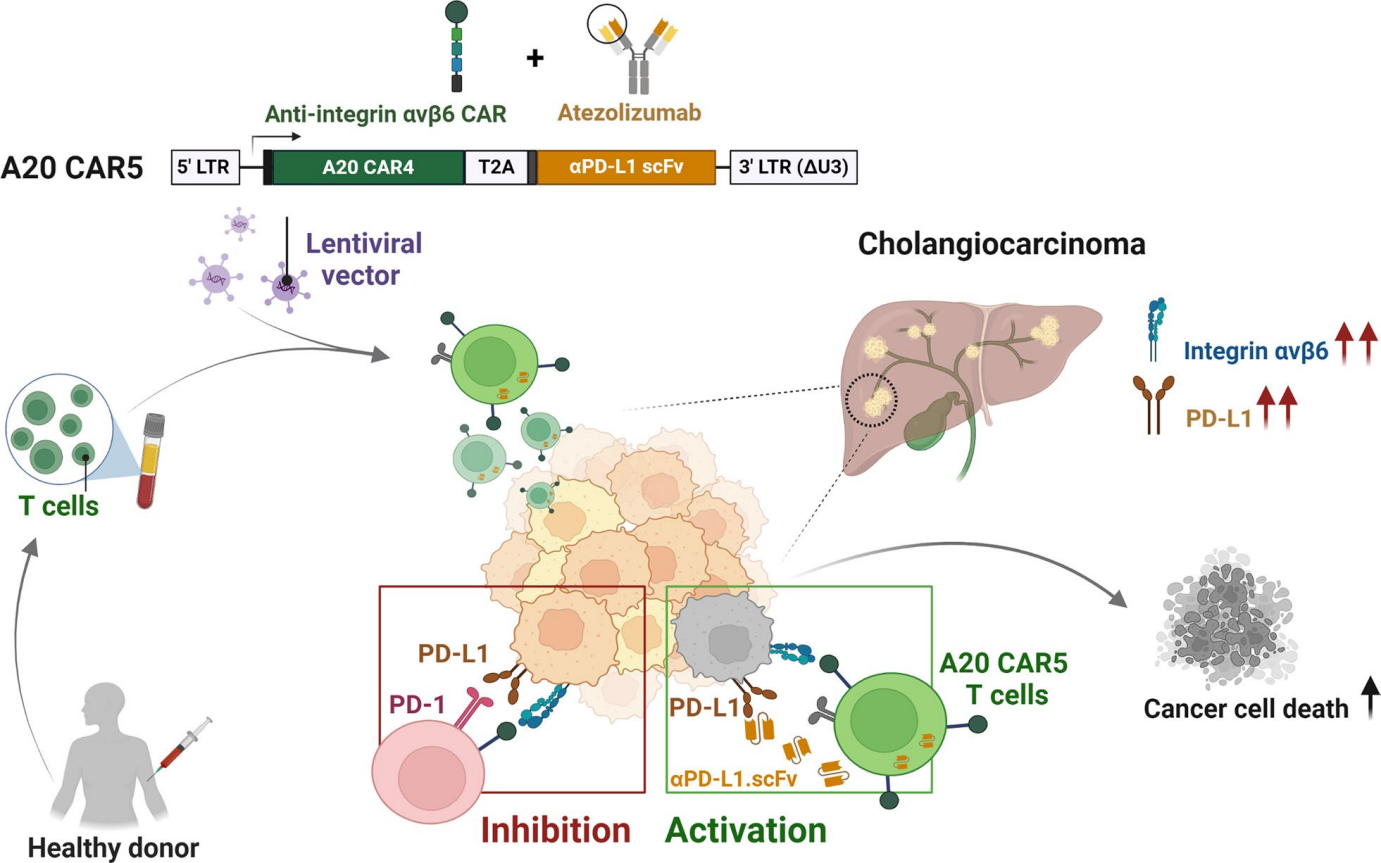
Schematic illustrating the mechanism of A20 CAR5 T cells, designed to mimic the natural activation process of T cells. The integrin αvβ6-targeted CAR recognizes and binds to integrin αvβ6 present on cancer cells, while PD-L1, an immune checkpoint molecule, inhibits T cell activity by interacting with PD-1 expressed on the T cell surface. Full T cell activation occurs when integrin αvβ6 is recognized, and PD-L1 is neutralized by the secreted anti-PD-L1 scFv. Without this neutralization, PD-L1 remains active, preventing full T cell activation and reducing the immune response

In conclusion, our study demonstrates the promising potential of combining CAR T cell therapy targeting integrin αvβ6 with PD-L1 blockade to enhance anti-tumor activity in CCA. By engineering A20 CAR5 T cells to co-express A20 CAR4 and secrete anti-PD-L1 scFv, we achieved improved tumor cytotoxicity and functionality compared to conventional A20 CAR4 T cell. Our findings emphasize the importance of targeting the inhibitory PD-1/PD-L1 signaling axis to overcome immunotherapy resistance in solid tumors like CCA (Fig. 7). Through in vitro experiments and 3D spheroid culture assays, we provided valuable insights into the mechanisms governing CAR T cell function and persistence within the TME. This study represents a significant advancement in the development of effective immunotherapeutic strategies for CCA, with the ultimate aim of improving patient outcomes and prognosis.

However, our study has several limitations that should be acknowledged. Firstly, we did not investigate the expression levels of integrin αvβ6 and PD-L1 in patient-derived samples, which would provide critical insights into the clinical relevance of our approach in Thai patients. Additionally, the lack of clinical correlation limits our ability to predict the translational potential of A20 CAR5 T cells in a diverse patient population. Secondly, further investigations utilizing fixed and embedded tumor spheroids with IHC or immunofluorescence staining are necessary to achieve a more precise quantification of CAR T cell infiltration. In the current study, we have addressed this aspect by performing a detailed reanalysis of infiltration depth and T cell numbers, providing valuable insights into the distribution and penetration of CAR T cells within the spheroids. Finally, our findings are based on in vitro models, and further validation in in vivo CCA models is essential to assess the long-term efficacy, safety, and therapeutic potential of A20 CAR5 T cells in a more complex tumor microenvironment.

Future research could expand on these findings by investigating the therapeutic potential of A20 CAR5 T cells. Exploration of the mechanisms responsible for the synergistic effects observed with integrin αvβ6 targeting and PD-L1 blockade could reveal new therapeutic targets and strategies for enhancing CAR T cell therapy in CCA. Examining the potential benefits of combining A20 CAR5 T cells with other immunomodulatory agents or treatment modalities, such as chemotherapy or targeted therapy, may overcome limitations associated with monotherapy. Finally, clinical trials assessing the safety, efficacy, and tolerability of A20 CAR5 T cells in CCA patients will be essential for translating these preclinical findings into clinical practice and improving patient outcomes.

## Supplementary Information

Additional file 1.

Additional file 2.

Additional file 3.

Additional file 4.

## Data Availability

Not applicable.
