# Supplementary figures and images for: Enhanced cytotoxicity against cholangiocarcinoma by fifth-generation chimeric antigen receptor T cells targeting integrin αvβ6 and secreting anti-PD-L1 scFv
Source: J Transl Med. 2025 Apr 16;23:451. doi: 10.1186/s12967-025-06453-y (PMC12004729; doi:10.1186/s12967-025-06453-y)

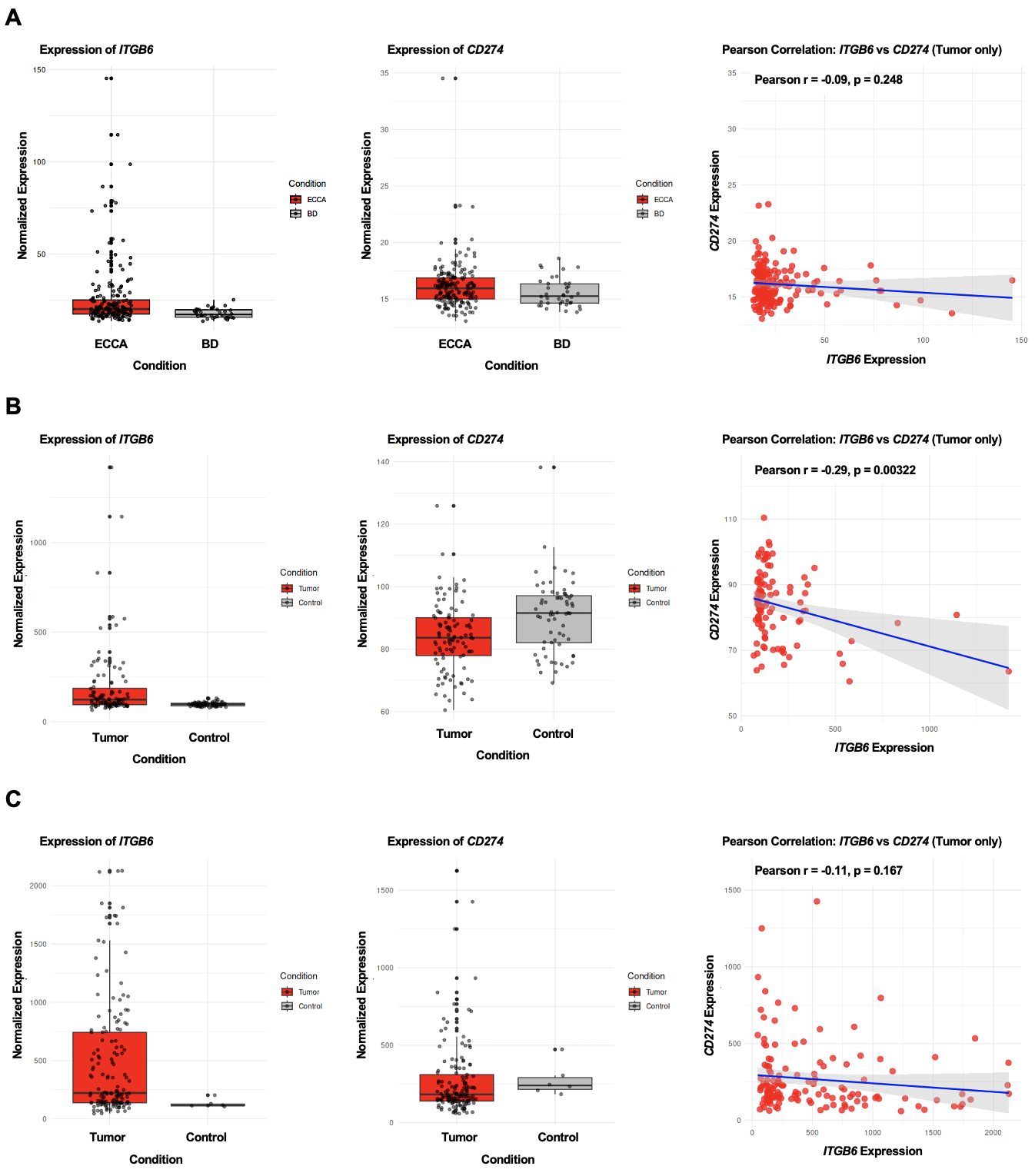

Supplement: Supplementary file 1 — Additional file 1. [file 12967_2025_6453_MOESM1_ESM.tif]

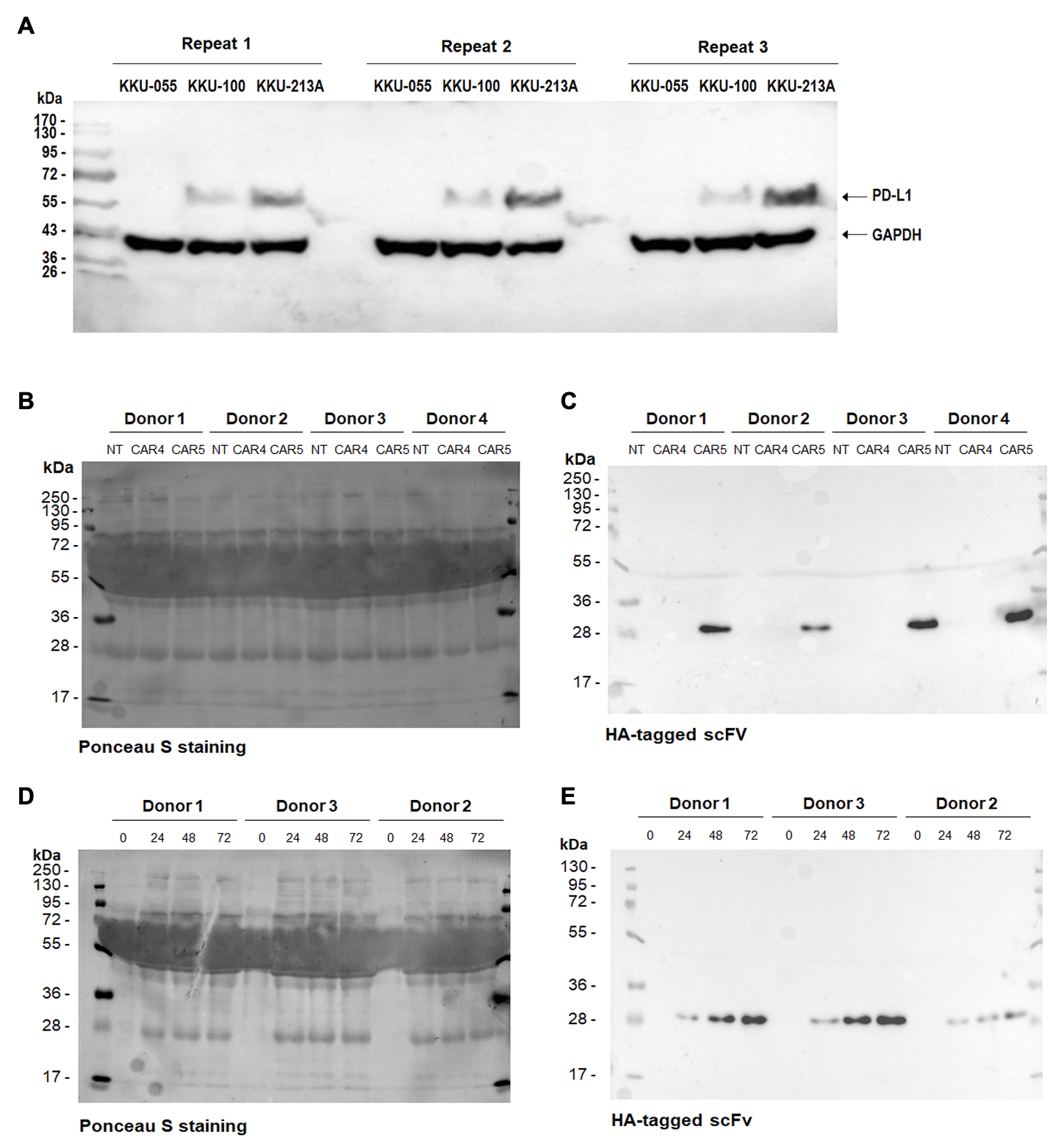

Supplement: Supplementary file 2 — Additional file 2. [file 12967_2025_6453_MOESM2_ESM.tif]

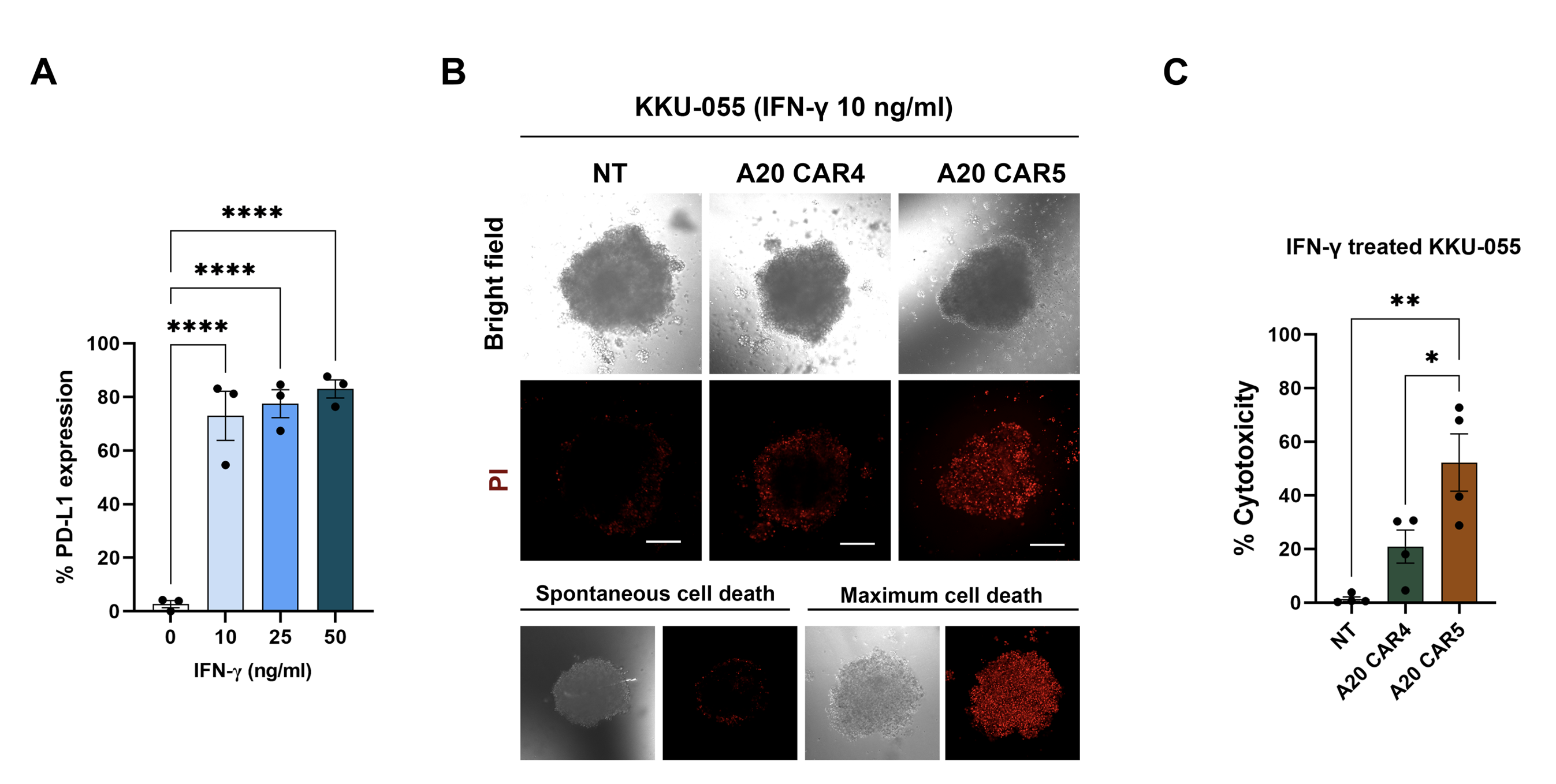

Supplement: Supplementary file 3 — Additional file 3. [file 12967_2025_6453_MOESM3_ESM.tif]

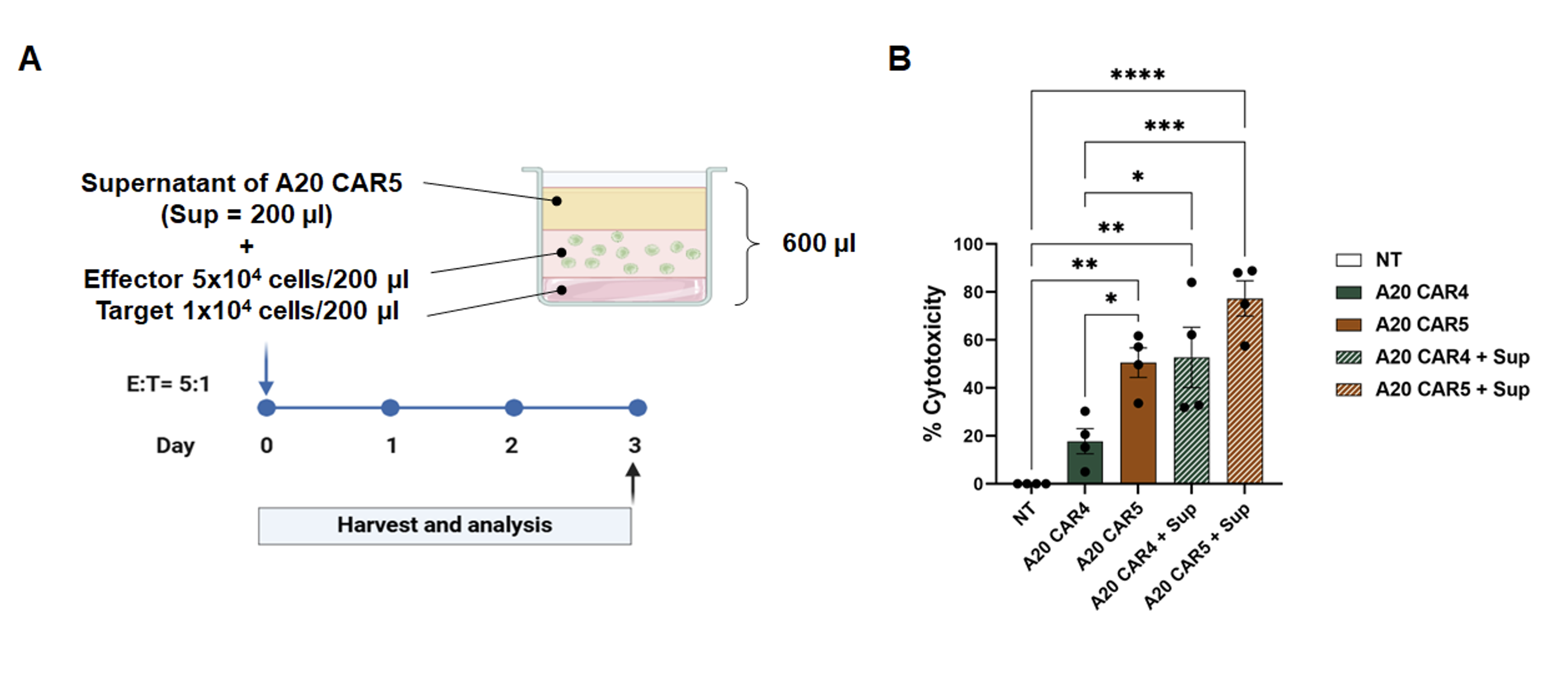

Supplement: Supplementary file 4 — Additional file 4. [file 12967_2025_6453_MOESM4_ESM.tif]
